# Comparative Analysis of the Flexural Stiffness of Pinniped Vibrissae

**DOI:** 10.1371/journal.pone.0127941

**Published:** 2015-07-01

**Authors:** Carly C. Ginter Summarell, Sudeep Ingole, Frank E. Fish, Christopher D. Marshall

**Affiliations:** 1 Department of Wildlife and Fisheries Sciences, Texas A&M University, College Station, Texas, 77843, United States of America; 2 Department of Marine Engineering Technology, Texas A&M University, Galveston, Texas, 77553, United States of America; 3 Department of Biology, West Chester University, West Chester, Pennsylvania, 19383, United States of America; 4 Department of Marine Biology, Texas A&M University, Galveston, Texas, 77553, United States of America; Sonoma State University, UNITED STATES

## Abstract

Vibrissae are important components of the mammalian tactile sensory system and are used to detect vibrotactile stimuli in the environment. Pinnipeds have the largest and most highly innervated vibrissae among mammals, and the hair shafts function as a biomechanical filter spanning the environmental stimuli and the neural mechanoreceptors deep in the follicle-sinus complex. Therefore, the material properties of these structures are critical in transferring vibrotactile information to the peripheral nervous system. Vibrissae were tested as cantilever beams and their flexural stiffness (*EI*) was measured to test the hypotheses that the shape of beaded vibrissae reduces *EI* and that vibrissae are anisotropic. *EI* was measured at two locations on each vibrissa, 25% and 50% of the overall length, and at two orientations to the point force. *EI* differed in orientations that were normal to each other, indicating a functional anisotropy. Since vibrissae taper from base to tip, the second moment of area (*I*) was lower at 50% than 25% of total length. The anterior orientation exhibited greater *EI* values at both locations compared to the dorsal orientation for all species. Smooth vibrissae were generally stiffer than beaded vibrissae. The profiles of beaded vibrissae are known to decrease the amplitude of vibrations when protruded into a flow field. The lower *EI* values of beaded vibrissae, along with the reduced vibrations, may function to enhance the sensitivity of mechanoreceptors to detection of small changes in flow from swimming prey by increasing the signal to noise ratio. This study builds upon previous morphological and hydrodynamic analyses of vibrissae and is the first comparative study of the mechanical properties of pinniped vibrissae.

## Introduction

Many organisms have developed mechanosensory structures to detect physical cues in their environment. Often, these structures are small, hair-like and abundant. For example, some freshwater insects use such structures to detect benthic flow patterns in streams [1,2] and marine copepods use hair-like structures called setae to detect small hydrodynamic signals at depth and near the sea surface [3–7]. The smallest filiform hairs found on insects and crustaceans are some of the most discriminatory sensory organs in the animal kingdom, operating on a microscopic level to detect water particle displacement [8]. Among aquatic vertebrates, the lateral line system of fishes is among the best studied mechanosensory systems [9–13]. The lateral line system allows an individual to receive information regarding the flow regime around its body that may originate from conspecifics (for schooling), predators, prey or itself, as well as other biological and abiotic cues (i.e., currents) in its environment [10,14,15]. The lateral line contains neuromast organs that transduce fluid forces to mechanoreceptors, hair cells [10,16,17]. Differences in the morphology of head and trunk canals of the lateral line have been correlated with differences in ecological factors such as habitat, swimming style and schooling behavior [12].

Vibrissae (whiskers) are modified hairs of mammals that are used as sensory organs. Vibrissae are comprised of a blood filled follicle-sinus complex (F-SC) that is heavily innervated by cranial nerve V, which courses through the F-SC to terminate on a variety of mechanoreceptors. The mystacial vibrissae, found on both sides of the muzzle, are the largest group. Among mammals, vibrissae are largest in pinnipeds [18,19] and as in other mammals, their mystacial vibrissae are arranged in an array of columns and rows. The vibrissae can be protruded into the water flow around their body, which allows them to navigate their aquatic environment and even track biogenic hydrodynamic trails in the water using this sense alone [20–26] and reviewed by [27]. Traditionally, the hair shafts of pinniped vibrissae have been classified as smooth or beaded, with a repeating sequence of crests and troughs to give a sinusoidal profile along the length [27–30]. Most species have a solid but elliptical cross-section [31]. The sinusoidal wavelength is on the order of millimeters and the greater and smaller diameters of the cross-section are out of phase by approximately 180 degrees. All phocid (true or earless) seals, with the exceptions of monk (*Monachus* spp.) and bearded (*Erignathus barbatus*) seals, show this distinct beaded profile [28–30, 32–36]. The vibrissae of ringed (*Pusa hispida*) and harbor (*Phoca vitulina*) seals have been observed to vibrate during swimming due to vortex shedding [21,33], and this is likely true for all phocid whiskers. The beaded morphology of phocid seal vibrissae decreases these vibrations during swimming compared to smooth vibrissae [30,37] but see [31]. This reduction in vibration likely increases the signal to noise ratio of vibrotactile stimuli and enhances the detection capability of mechanoreceptors in the underlying hair follicles [30,37,38]. When actively hunting, seals protract their vibrissae, which then vibrate at a certain frequency based on their mechanical properties [21]. Only recently has the morphology of the hair shaft, particularly the impact of the beaded morphology on water flow [30,31,37] and the diversity of morphologies among phocids [29,32] been investigated.

It is clear that pinnipeds’ vibrissal hair shafts are modulating environmental signals via changes in the cross-sectional shape and morphology of the beaded sinusoidal profile. These properties modify how information is transferred to the mechanoreceptors deep in the F-SC. Therefore, the material properties of vibrissal hair shafts are likely crucial to the transmission of information. Changes in mechanical properties likely interact with the diversity of hair shaft morphology to affect flexural stiffness and modulate the amplitude and frequency of vibrotactile cues that arrive at the F-SC mechanoreceptors. In this way, the hair shafts of pinniped whiskers, and likely all mammals, function as a biomechanical filter [39]. Such ideas are not new. Dykes [18] postulated that the increased response of seal vibrissae to high frequency stimuli was related to hair shaft mechanical properties. However, the mechanical properties of vibrissal hair shafts (hereafter called vibrissae) are currently unexplored.

In this study, pinniped vibrissae were tested as simplified cantilever beams projecting from the muzzles of seals and engineering beam theory was used to investigate mechanical differences among a variety of pinnipeds. In beam theory, when a cantilevered beam is bent downward, the top half of a beam is placed in tension while the lower half of the beam is placed in compression [40]. A neutral plane running longitudinally through the center of the beam experiences no tensile or compressive stresses [40,41] during bending. A beam’s resistance to bending depends upon both the material of which it is made and the arrangement of that material around the neutral plane. Vibrissae exhibit specific orientation with an ellipsoidal cross-section, and are curved and tapered along their length. Calculation of bending forces, such as those generated when vibrissae are subjected to water flow, requires knowledge of both the mechanical properties and the geometric arrangement of the material, also known as the second moment of area (*I*). The measure of a material’s elasticity is referred to as Young’s modulus (*E*). Flexural stiffness is the product of Young’s modulus and the second moment of area (*EI*) [41–43]. Young’s modulus is a material property and is therefore assumed to remain constant over the length of a structure [41]. However, *I* depends upon the cross-sectional area of the structure and this geometry can vary over the length of the beam and be modified by the organism’s developmental plan. In strict terms, measures of *E* are only valid if structures are homogeneous, isotropic, linearly elastic (Hookean), deform equally under both tensile and compressive forces, and deform less than 10% when loaded [40,41]. Obviously many biological materials violate some of these assumptions, but a good estimate of *EI* can be calculated if both the Young’s modulus and the second moment of area can be measured [44,45]. Due to the depth of knowledge regarding the function of pinniped vibrissae in terms of the neurobiology, F-SC microanatomy, hair shaft morphology, computational fluid dynamics, and numerous behavioral studies, the vibrissae of pinnipeds provide an interesting model system in which to investigate the influence of material properties on sensory function. We predict that, as found in vibrissal hair shaft morphologies [29,31,32], there will be a diversity of flexural stiffness values among pinniped species that likely influences their function. The first obvious morphological difference among pinniped vibrissae is whether they are beaded or smooth. It has been postulated that the beaded profile possessed by most phocids increases the stiffness of the “sensory lever” of these vibrissae [46]. However, an alternative hypothesis is that beaded morphology decreases vibrissal hair shaft stiffness relative to smooth whiskers since the distribution of the material away from the neutral axis will vary and on average be closer to the neutral axis than smooth vibrissae (if the greatest diameters between smooth and beaded whiskers are kept constant). Therefore, the objective of this study was to measure flexural stiffness (*EI*) for vibrissae from numerous pinniped species to test the hypothesis that traditionally beaded vibrissae exhibit a lower *EI* compared to smooth vibrissae. In addition, since most pinniped whiskers have an elliptical cross-section and are curved along their length, we tested the hypothesis that flexural stiffness was anisotropic in vibrissae, depending upon orientation.

## Materials and Methods

### Ethics Statement

Whole mystacial vibrissal pads were collected from dead animals by stranding networks in New Jersey, New England and California and from legal, indigenous hunts in Alaska, and single vibrissae were collected opportunistically when shed by captive animals. IACUC approval was not required since only dead animals or naturally shed vibrissae were utilized. All samples were collected under a National Marine Fisheries Service (NMFS) Southeast Regional Office salvage permit letter to CDM and NMFS permits #358–1585 and 358–1787 issued to the Alaska Department of Fish and Game. The sample collection procedure was reviewed as part of obtaining the salvage permit letter.

### Test Samples

**Fig 1:**
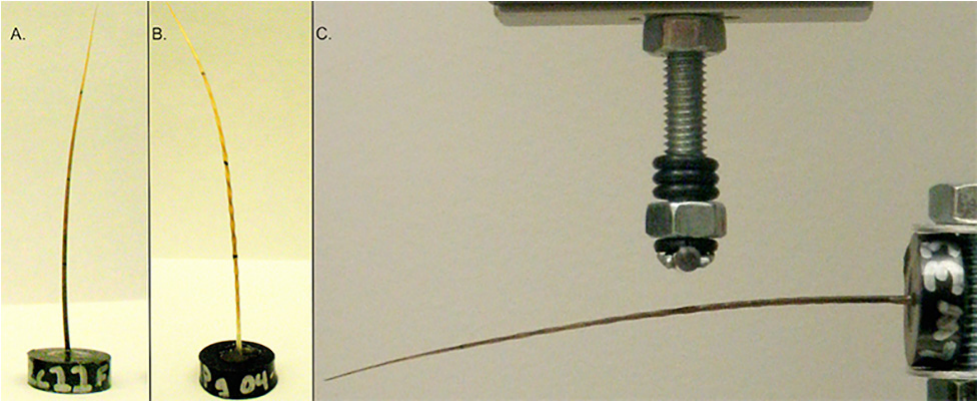
Photographs of vibrissae prepared for testing. A) a California sea lion vibrissa potted in epoxy mold, B) a harp seal vibrissa potted in epoxy mold, and C) the MTS Insight apparatus testing set up with a Weddell seal vibrissa potted in epoxy mold and held horizontally for cantilever bending. The major axis of each vibrisse is facing the reader and the minor axis is perpendicular to the page.

Mystacial vibrissae from 11 pinniped species and 43 individuals were tested (Table 1). Seven of the pinnipeds were from the family Phocidae and the remaining four species were from the Otariidae. Beaded vibrissae were present in the phocids, with the exception of bearded seals (*Erignathus barbatus*). Bearded seals and all of the otariids possessed smooth vibrissae. Since there may be variation within individual pinnipeds depending on the size of the vibrissa and its location on the muzzle, the longest vibrissae, which tended to be found in the lower rows and posterior columns, were chosen from the same location on the vibrissal array across species to standardize comparisons. Although the exact location of shed vibrissae could not be known, the shed vibrissae were consistent with the largest mystacial whiskers from other pinnipeds, where their exact location is known, and their material properties were in line with vibrissae collected in these controlled methods. Morphological analyses using vibrissae from many of the same individuals tested in the present study showed no significant differences in vibrissal length or area among age classes or between genders [32]. Vibrissae were removed from the muzzle by cutting them off at the skin surface, external to the follicle. Mechanical properties of the vibrissae were tested using a MTS Insight 5 SL uniaxial load frame (MTS Systems Corporation, Eden Prairie, MN, USA). A 25N load cell (MTS Systems Corporation, Eden Prairie, MN, USA) was equipped with a circular horizontal pin (shown above the vibrissa in Fig 1C) that could roll slightly to create a near frictionless contact during point force loading.

**Table 1:** Material testing vibrissae samples.

Vibrissal Profile	Family	Species	Number of Individuals
Beaded	Phocidae	Gray seal (*Halichoerus grypus* Fabricius, 1791)	5
	Phocidae	Harbor seal (*Phoca vitulina* Linnaeus, 1758)	5
	Phocidae	Harp seal (*Pagophilus groenlandicus* Erxleben, 1777)	5
	Phocidae	Ringed seal (*Pusa hispida* Schreber, 1775)	5
	Phocidae	Spotted seal (*Phoca largha* Pallas, 1811)	5
	Phocidae	Weddell seal (*Leptonychotes weddellii* Lesson, 1826)	3
Smooth	Phocidae	Bearded seal (*Erignathus barbatus* Erxleben, 1777)	5
	Otariidae	California sea lion (*Zalophus californianus* Lesson, 1828)	5
	Otariidae	Guadalupe fur seal (*Arctocephalus townsendi* Merriam, 1897)	1
	Otariidae	Northern fur seal (*Callorhinus ursinus* Linnaeus, 1758)	2
	Otariidae	South American fur seal (*Arctocephalus australis* Zimmermann, 1783)	2

The number of individuals is given, classified by vibrissal profile, family and species.

### Testing Procedure and Equations

Samples of vibrissae were secured at the base in plastic molds filled with epoxy to keep the base stationary during testing (Fig 1). Vibrissae were pushed into the epoxy so that the base was flush with the bottom of the mold during potting. A customized apparatus was fitted into the lower grip of the MTS to hold each vibrissa horizontally. A percentage of the length of vibrissae was used to standardize the testing location among all species. The distance along the vibrissa from the epoxy mold to the point force load was used as the length of the beam (*L*; Eqs 1 and 2). A point force was applied at 25% and 50% of the total length of the vibrissa base. This length was calculated by:

$$L=\left(L_{o}-H_{epox}\right)\;\times \;0.25,$$ (1)

and

$$L=\left(L_{o}-H_{epox}\right)\;\times \;0.50,$$ (2)

where L is the length of the vibrissa (mm), Lo is the overall vibrissa length (mm), and Hepox is the height of the epoxy base (mm).

The applied force and deflection were recorded using the MTS data acquisition software and *E* was calculated from the linear (elastic) portion of the load-extension curve where the material is Hookean, i.e., extension is proportional to force [47]. The slope of the linear portion of the graph was calculated by the equation for a line:

Y=mx+b, (3)

where *Y* is the applied force (*F)* and x is deflection *(δ)*. The y-intercept (*b*) is zero since the curve starts at the origin.

*EI* is calculated by [48]:

$$EI=\frac{FL^{3}}{3\delta }\;,$$ (4)

where *F* is the applied force, *L* is the effective beam length, or distance from the point of attachment to the point force, and δ is the deflection (the change in length divided by the original length). Through simple algebraic substitution and transposition of elements from Eqs 3 and 4, *E* can be calculated as:

$$E=m\;\times \;\frac{L^{3}}{3I},$$ (5)

where *E* is the elastic modulus, *m* is the slope of the linear portion of the force-extension curve, and *I* is the second moment of area of the vibrissae.

We approximated the shape of the vibrissae as a cylinder. The actual shape of pinniped vibrissae is conical [49], but its taper is very small at the locations we tested. Therefore, the error due to assuming it to be cylindrical will also be negligible. The shape and beaded structure influence the value of I, and consequently influence flexural stiffness.

Vibrissae were bent in the anterior-posterior orientation, then rotated and bent in the dorsal-ventral orientation. The major and minor axes (*w* and *h*) of the ellipsoidal cross section of each vibrissa were measured with digital calipers (Mitutoyo Corporation, Kawasaki, Japan). Flexural stiffness measurements were collected at 25% and 50% of the length of each vibrissa from the base. The equation of *I* for an ellipse with radii *w* and *h* was used [40,41]:

$$I=\frac{\pi wh^{3}}{4},$$ (6)

$$I_{anterior}=\frac{\left(\pi w^{3}h\right)}{4},$$ (7)

$$I_{dorsal}=\frac{\left(\pi wh^{3}\right)}{4},$$ (8)

where *w* is the radius of the major axis and *h* is the radius of the minor axis.

The equations above were used to calculate *EI* in both the dorsal and anterior orientations of bending.

### Mechanical Testing Criteria

The test conditions were optimized using several criteria during data collection and analysis. The slip of the sample during the initial phase of the test was determined from the raw load-extension data and thus the beginning of the test was determined. This slippage can occur due to improper gripping of the plastic molds or minor tolerance in the thread of the vertical screw. This can easily be detected. In these experiments, the grips were not specified by the test standards so they were designed to suit to the test configurations. However, these slippages were negligible. The test was determined to have begun when the load value and the extension value were positive and linearly proportional. To determine the effect of the crosshead speed (rate of application of load) on *EI*, beaded vibrissae and smooth vibrissae were tested at nine different test speeds ranging from 0.5mm/min to 5mm/sec. One-Way ANOVA determined that the speed of the crosshead did not affect the material properties of either beaded or smooth vibrissae (P = 1.00). These results are in agreement with a previous study on the compressive modulus of keratinous horse hooves, which compared speeds from 10–100 mm/min and found no significant effect [50]. Based on the lack of difference among speeds observed in this study, a testing speed of 10mm/min was used throughout the rest of the trials. This speed was intermediate among those tested and decreased the time necessary for each trial.

**Fig 2:**
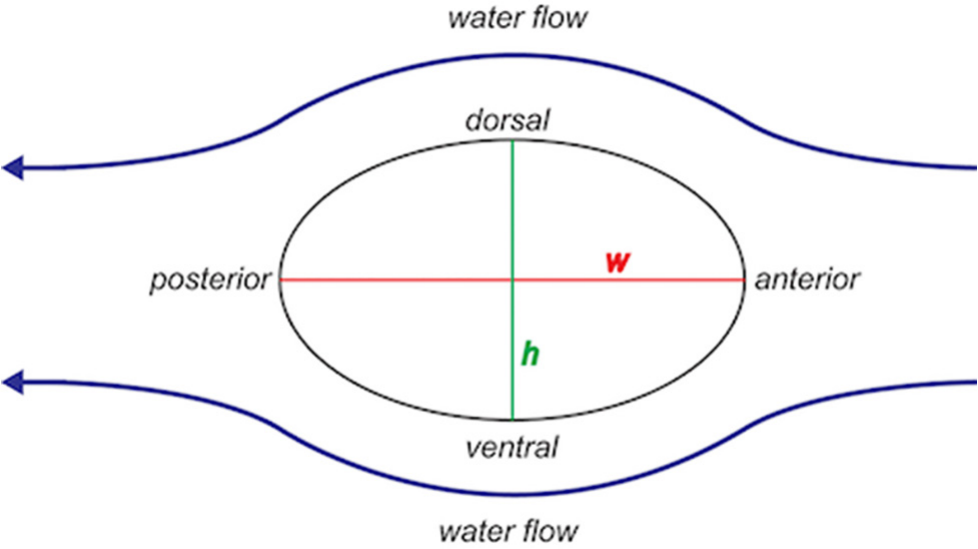
Diagram of vibrissal orientation in water flow and testing orientations.

One vibrissa from each of five species with beaded hairshafts and two species with smooth hairshafts were tested in the four major orientations: anterior, posterior, dorsal and ventral. Morphologically, as a seal swims through water, flow crosses the long cross-sectional axis of each vibrissa (Fig 2), that is, the major axis (*w*). Accordingly, this orientation was denoted as the anterior orientation, and the orientation 180 degrees from the anterior orientation was denoted as the posterior orientation to create an anterior-posterior orientation (A-P). The convex side of each vibrissa was designated as the dorsal orientation and the concave side (180 degrees from the dorsal surface) was denoted as the ventral orientation,for both phocids and otariids. This dorsal-ventral (D-V) orientation corresponds to the minor axis (*h*) of the vibrissal cross-section (Fig 2). These orientations were confirmed by the inspection of whole muzzles containing the entire mystacial array of vibrissae for numerous species, as well as in live animals (personal observation, CDM). Another subset of vibrissae was used to test the hypothesis that EI was invariant regardless of whether vibrissae were wet or dry. This was important to translate the laboratory work to vibrissal function in live animals. Vibrissae were tested (in both the anterior-posterior and dorsoventral orientations) after being stored dry and then were placed in distilled water for 20 min. Excess water was wiped off with a paper towel and the vibrissae were immediately tested again in both orientations. In all testing scenarios, five consecutive trials were run in each orientation with the load completely removed from the vibrissa for one minute between trials.

### Statistical Analysis

Variation in *EI* measurements among species and between orientations was presented as means ± one standard deviation (s.d.) and assessed using ANOVA with species or orientation as the independent variable and EI as the dependent variable, followed by Tukey HSD post-hoc tests in JMP software (v. 8.0.1, SAS Institute, Inc., Cary, NC, USA). Fur seals (*Arctocephalus australis*, *Arctocephalus townsendi*, *Callorhinus ursinus*) were pooled as one group for statistical analysis due to the low number of individuals per species. All data were log transformed to obtain normality before statistical testing. Results were determined to be statistically significant at P<0.05.

## Results

**Fig 3:**
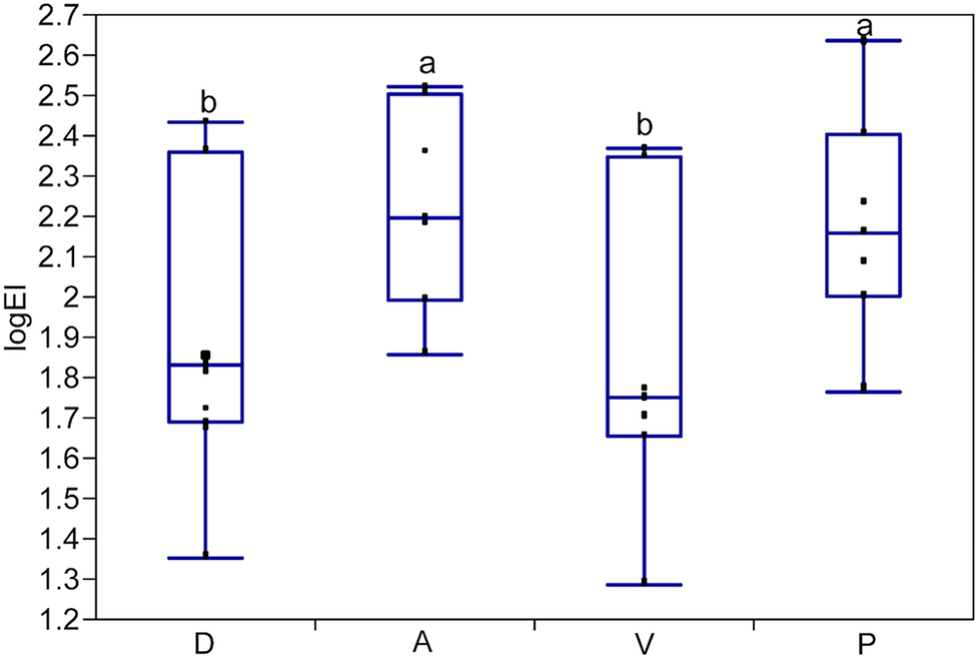
Results of materials testing in four orientations. A subset of pinniped vibrissae was tested in all four orientations. D = Dorsal, A = Anterior, V = Ventral, P = Posterior. Dorsal and ventral did not differ from each other but had significantly lower mean flexural stiffness values than anterior and posterior, which also did not differ from each other. Different letters indicate significant differences between orientations.

Flexural stiffness values for samples tested in the anterior and posterior orientations were not significantly different from each other, nor were the values for samples tested in the dorsal and ventral orientations (Fig 3). However, both anterior and posterior orientations of vibrissae significantly differed in *EI* (P = 0.001; Fig 2) from samples tested in the dorsal and ventral orientations. This makes sense given that the A-P orientation corresponds to the major axis of each vibrissae and the D-V orientation corresponds to the minor axis of each vibrissa. Since *EI* differed in only two orientations (A-P vs. D-V), only the anterior and dorsal orientations were tested within and among species. Prior testing demonstrated that *EI* values of dry *versus* wet trials did not significantly differ from one another. Values of all species and individuals demonstrated that *EI* values of vibrissae were anisotropic. Vibrissal *EI* values in the A-P orientation were 1.5 to 2.9 times greater than the D-V orientation (P<0.001) at the 25% testing length and 1.4 to 3.4 times greater than the D-V orientation (P<0.001) at the 50% testing length (Table 2).

**Table 2:** Results of *EI* measurements at two locations on the vibrissae.

Vibrissal Profile	Species	25% *EI* (N/mm^2^)		50% *EI* (N/mm^2^)	
		A (Major)	D (Minor)	A (Major)	D (Minor)
Beaded					
	Gray	98.7 ± 32.36	34.4 ± 8.78	95.8 ± 27.01	27.9 ± 6.31
	Harbor	136.5 ± 38.38	68.5 ± 25.88	120.8 ± 35.65	49.9 ± 24.11
	Harp	152.7 ± 48.05	87.1 ± 44.22	118.4 ± 36.84	53.6 ± 29.22
	Ringed	81.2 ± 44.72	40.1 ± 17.89	71.7 ± 42.37	30.8 ± 13.97
	Spotted	159.1 ± 99.42	91.8 ± 59.51	143.7 ± 100.04	72.6 ± 47.47
	Weddell	287.6 ± 23.10	216.5 ± 10.73	248.9 ± 26.44	180.1 ± 34.94
Smooth					
	Bearded	434 ± 154.96	164 ± 79.41	371 ± 154.55	146.5 ± 66.31
	CA Sea Lion	278.8 ± 73.52	171.5 ± 83.70	264.7 ± 113.79	134.3 ± 88.11
	Fur Seals	590.1 ± 301.05	390.8 ± 229.42	559.7 ± 326.52	319.4 ± 231.53

Mean ± s.d. for *EI* is given for each species of pinniped analyzed in this study. A = anterior orientation and D = dorsal orientation.

Comparatively, *EI* varied among species. In the A-P orientation, mean *EI* ranged from 81.2 N/mm^2^ (for a ringed seal) to 590.1 N/mm^2^ (for a fur seal) at the 25% testing length, and from 71.7 N/mm^2^ (for a ringed seal) to 559.7 N/mm^2^ (for a fur seal) at the 50% testing length (Table 2). In the D-V orientation, mean *EI* values ranged from 34.4 N/mm^2^ (for a gray seal) to 390.8 N/mm^2^ (for a fur seal) at 25% testing length, and from 27.9 N/mm^2^ for a gray seal to 319.4 N/mm^2^ for a fur seal at 50% testing length (Table 2).

**Fig 4:**
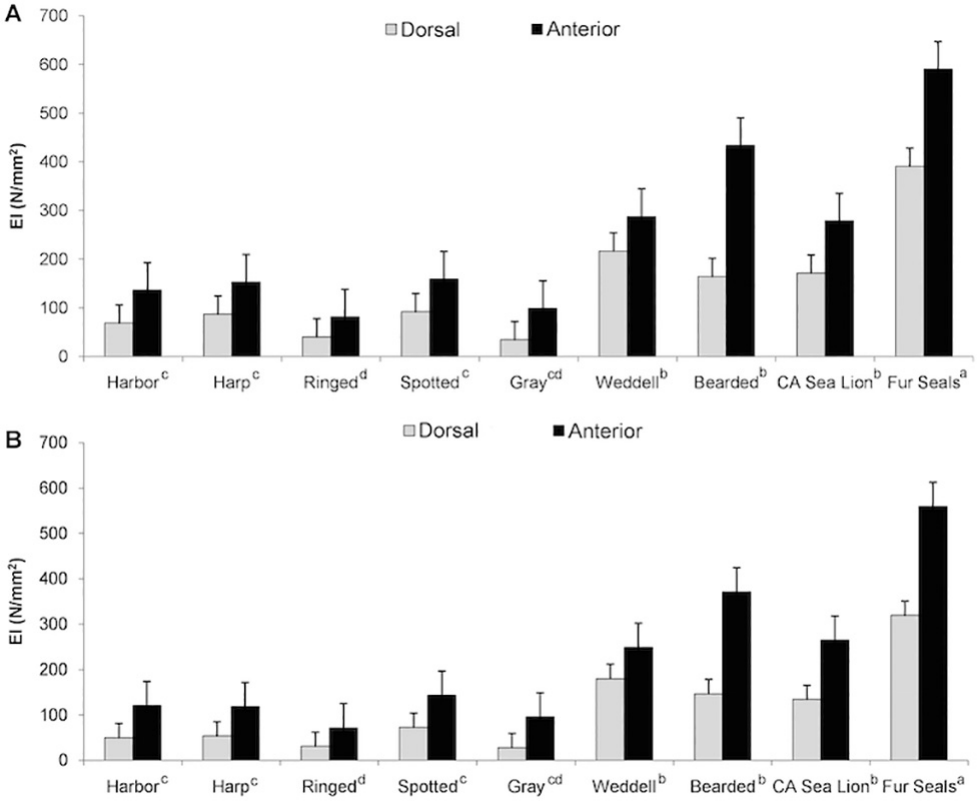
Results of *EI* measurements. A) 25% of the length and B) 50% of the length. Mean ± s.e.m. values are given for six species of pinnipeds with beaded vibrissae and three species with smooth vibrissae. D = Dorsal orientation and A = Anterior orientation. Species with different letters were significantly different.

At the 25% testing length, ringed seals had the lowest *EI* values in both the A-P and D-V orientations, and significantly differed from all species except gray seals (P<0.001). Fur seals had the highest *EI* values and significantly differed from all other species (P = 0.018; Fig 4A). The greatest difference between the A-P and D-V orientations was observed in gray seals and the smallest difference was observed in Weddell seals (Fig 4A). The five trials within each species in each orientation were not significantly different from each other.

At the 50% testing length, gray seals had the lowest *EI* values in the D-V orientation and ringed seals had the lowest *EI* values in the A-P orientation. Ringed seals were significantly different from all species except gray seals (P<0.001; Fig 4B). Fur seals had the highest *EI* values and significantly differed from all other species (P<0.001; Fig 4B). Once again, the greatest difference between the A-P and D-V orientations was observed in gray seals and the smallest difference was observed in Weddell seals (Fig 4B). The five trials within each species in each orientation were not significantly different from each other.

At the 25% testing length, bearded seals had the lowest E values in both the D-V and A-P orientations. Harp seals had the highest E value in the D-V orientation and CA sea lions had the highest E value in the A-P orientation (Table 3). Bearded seals also had the lowest E values in both orientations at the 50% testing length. Fur seals had the highest E values in both orientations at the 50% testing length (Table 3).

**Table 3:** Values of Young’s Modulus (E).

Species	25%		50%	
	D-V (Minor)	A-P (Major)	D-V (Minor)	A-P (Major)
Gray	8.15 ± 2.47	5.92 ± 1.76	16.47 ± 3.20	8.71 ± 2.12
Harbor	11.96 ± 1.69	7.53 ± 2.38	22.87 ± 6.99	12.07 ± 1.89
Harp	15.84 ± 4.21	7.62 ± 2.86	25.27 ± 5.29	11.69 ± 4.85
Ringed	12.06 ± 3.14	7.88 ± 2.82	26.53 ± 6.51	16.97 ± 8.49
Spotted	11.94 ± 1.72	5.97 ± 0.96	18.22 ± 5.46	7.26 ± 1.41
Weddell	8.97 ± 2.65	7.02 ± 1.05	16.37 ± 6.39	11.56 ± 2.31
Bearded	6.96 ± 1.81	4.85 ± 0.98	8.63 ± 2.62	5.98 ± 0.94
CA Sea Lion	12.23 ± 1.52	14.10 ± 5.87	20.77 ± 4.96	15.99 ± 1.96
Fur Seals	11.90 ± 4.06	8.94 ± 2.85	29.05 ± 10.22	23.28 ± 7.86

Mean E values (GPa) ± standard deviation are presented by species, testing location and orientation.

Ringed seals had the smallest diameter and I value in both the major and minor axes at 25% and 50% of the total vibrissae length. Bearded seals had the largest diameter and I value in the major axis and fur seals had the largest diameter and I value in the minor axis at 25% of the total vibrissa length (Table 4). Bearded seals had the largest diameter and I values in the major axis and were equal to Weddell seals’ diameter in the minor axis at 50% of the total vibrissa length. Bearded seals also had the highest I values in the minor axis at 50% of the total vibrissa length (Table 4).

**Table 4:** Values of I and diameter.

Species	25%				50%			
	Diameter A-P (Major)	Diameter D-V (Minor)	I A-P (Major)	I D-V (Minor)	Diameter A-P (Major)	Diameter D-V (Minor)	I A-P (Major)	I D-V (Minor)
Gray	0.91 ± 0.12	0.46 ± 0.04	0.018 ± 0.008	0.004 ± 0.001	0.88 ± 0.14	0.34 ± 0.03	0.012 ± 0.006	0.002 ± 0.001
Harbor	0.91 ± 0.07	0.50 ± 0.11	0.018 ± 0.003	0.006 ± 0.003	0.81 ± 0.07	0.38 ± 0.09	0.010 ± 0.004	0.002 ± 0.002
Harp	0.96 ± 0.11	0.48 ± 0.06	0.022 ± 0.010	0.006 ± 0.003	0.85 ± 0.11	0.36 ± 0.05	0.011 ± 0.004	0.002 ± 0.001
Ringed	0.78 ± 0.21	0.44 ± 0.08	0.012 ± 0.010	0.003 ± 0.002	0.64 ± 0.17	0.33 ± 0.07	0.005 ± 0.004	0.001 ± 0.001
Spotted	0.97 ± 0.24	0.51 ± 0.12	0.028 ± 0.023	0.008 ± 0.006	0.93 ± 0.28	0.42 ± 0.10	0.022 ± 0.017	0.004 ± 0.004
Weddell	1.02 ± 0.03	0.80 ± 0.08	0.042 ± 0.008	0.026 ± 0.008	0.88 ± 0.04	0.65 ± 0.11	0.022 ± 0.006	0.013 ± 0.006
Bearded	1.37 ± 0.16	0.69 ± 0.10	0.093 ± 0.045	0.024 ± 0.012	1.24 ± 0.20	0.65 ± 0.10	0.068 ± 0.045	0.018 ± 0.011
CA Sea Lion	0.97 ± 0.16	0.65 ± 0.12	0.032 ± 0.020	0.015 ± 0.009	0.86 ± 0.14	0.51 ± 0.09	0.018 ± 0.010	0.006 ± 0.004
Fur Seals	1.15 ± 0.14	0.82 ± 0.18	0.068 ± 0.044	0.037 ± 0.034	0.91 ± 0.12	0.61 ± 0.13	0.025 ± 0.014	0.012 ± 0.009

Mean ± SD I and diameter values are presented for each species in the major and minor axes and at 25% and 50% testing lengths.

## Discussion

Hydrodynamic reception of vibrotactile stimuli left by pinniped prey is a complex process that is mediated by the structure of the F-SC, the type, number and placement of mechanoreceptors and their innervation, the length and taper of the vibrissal hair shafts, and the morphology and material properties of those hair shafts. In this study, we showed that flexural stiffness of pinniped whiskers is anisotropic. Vibrissae were stiffer in the anterior-posterior orientation than the dorsal-ventral orientation. Our values of Young’s modulus reported in this study were comparable to those of rat vibrissae, which ranged from 3–7.36 GPa [51,52]. Young’s modulus of harp seal vibrissae was previously measured at 1.8–3.3 GPa [53].

It is clear that the cross-sectional geometry of pinniped vibrissae is of high importance since the differences in *I* (rather than *E*) between the two orientations resulted in different *EI* values. In the A-P orientation, vibrissae are wider than they are tall, so more material is located further away from the neutral axis, resulting in greater flexural stiffness, as expected [40,41]. This orientation makes sense functionally, because the major axis of the vibrissal cross-sectional ellipse is in-line with water flow past the individual [27,30]. *EI* in both orientations were similar at both 25% and 50% of vibrissal length in all species, and all species demonstrated higher *EI* values at 25% of the length than at 50% of the length, as expected. Vibrissae were stiffer at the base and became more flexible further from the base. However, the relative difference of EI is of interest as it may impact foraging tactics. Because hydrodynamic trail-following relies on vibrotactile stimuli being transmitted to the mechanoreceptors by the hair shaft, the testing location near the base of the vibrissae (i.e., 25% of total length) was likely more indicative of the mechanical function that allows these tracking behaviors. Studies on other biological materials have had difficulty obtaining data near the tip of the structure due to slippage [44]. Trials at 75% of the length of the vibrissae were abandoned in the present study for similar reasons. The mechanical properties of the tips of these structures may best be estimated using alternative methods such as Finite Element Modeling. A study of flexural stiffness in pigeon feathers demonstrated a similar trend, in which maximum values of *I* occurred at or near the insertion of the feather into the skin (between 0 and 20% of feather length) and then decreased along the length of the feather [54,55].

We hypothesized that a beaded profile of most phocid vibrissae would alter *I* in such a way as to reduce *EI* compared to the smooth profile of most otariid vibrissae. Our rationale behind this hypothesis was that the sinusoidal wavy appearance of beaded vibrissae should, on average, decrease the distance between the neutral axis of the beam and the majority of material relative to smooth vibrissae. The prediction that *EI* is lower in beaded hair shafts was supported by the results of this study. In general, otariid vibrissae were stiffer than phocid vibrissae, with the exception of Weddell seals. Because bearded seal vibrissae are also smooth, their greater flexural stiffness was consistent with the smooth vibrissae of otariids. In addition, bearded seals may have displayed higher *EI* values because their vibrissae are used in active touch behavior while foraging on benthos [56–58], rather than hydrodynamic trail following of fish, which is a divergent behavior from other species in this study. However, the flexural stiffness of beaded Weddell seal vibrissae was similar to smooth California sea lion vibrissae. Although it was reported that cross-sectional shape of pinniped vibrissae is elliptical [30,31], our data demonstrated that this property varied across species and impacted *EI* values. For example, Weddell seals in this study displayed a nearly circular cross-section, which minimized differences in flexural stiffness between the A-P and D-V orientations. Such a simple variable has functional consequences in prey detection during hydrodynamic trail following.

The fact that otariid vibrissae were stiffer than the beaded vibrissae of phocids may not seem intuitive at first because harbor seals (the best studied species in terms of sensory capabilities) also possess stiff whiskers. The finding that phocid vibrissae have lower EI values than otariid vibrissae was unexpected but currently the detailed mechanism of stimuli transmission is unknown. For information to be transmitted to the mechanoreceptors in the F-SC, the vibrissal hair shafts must be stiff enough to be able to be protruded and maintained in the water flow as the individual follows a hydrodynamic trail. However, the vibrissae must be flexible enough to allow information from vortex trails in the flow to be transmitted along the hair shaft to the follicle. It is possible that if the hair shaft is too stiff, the transmission of small vibrotactile cues may not be conveyed as effectively to the mechanoreceptors. Vibrissae not only function to transmit vibrotactile cues from the environment to the mechanoreceptors of the F-SC, but also amplify those signals. In laboratory rats (*Rattus rattus*), the natural resonance frequencies of vibrissae amplify tactile cues and modulate how vibrotactile cues are conveyed to the central nervous system [52,59,60]. Rat vibrissal hair shafts exhibited a resonance when brushed past an object in its environment. This resonance was dependent upon the morphology (length and width) of the vibrissae. Cross-sectional length and width of vibrissae (i.e., major and minor axes) are the main factors in calculating the second moment of area and flexural stiffness. Flexural stiffness of vibrissae will affect the natural resonances of each hair shaft and should be considered as a proxy for vibrissal natural resonances. In rats, the vibrissal array represents a resonance analyzer, with shorter vibrissae tuned to different resonances than longer vibrissae, which is analogous to the hair cells of cochlea in mammalian ears [52,59,60]. In addition, rats can change these biomechanical properties possibly by increased muscle tension or changes in blood flow (and pressure) to the F-SC [52,61,62]. We suggest that pinniped vibrissal hair shafts function similarly, but in the case of beaded phocid vibrissae, there is an interplay among natural resonance frequencies, sinusoidal morphology and hydrodynamic effects resulting from the sinusoidal morphology that further modulate environmental vibrotactile cues compared to otariids. Evidence supporting this hypothesis is provided by performance data from both harbor seals and California sea lions. Harbor seals have a higher success rate in tracking hydrodynamic trails than California sea lions [20–23]. The higher *EI* values observed in smooth vibrissae may make these structures less able to detect small deflections from changes in flow created by a swimming prey item. Additionally, the size of vibrissae varies on the muzzle, from about 1.5 cm to 10 cm long in phocids [29]. This range in size may result in a range of tuned frequencies accessible to the animal, which may vary among species. Additional mechanical testing on vibrissae of various sizes would help elucidate this possibility.

The pronounced differences in *EI* between the A-P and D-V orientations likely have sensory consequences. As the animal swims forward, the vibrissae vibrate at a frequency related to their swimming speed [33]. The vibrations are induced from the alternating generation of vortices that are shed from the vibrissal surface into the wake [30,38]. However, the angle of the vibrissa to the water flow also affects the vibration frequency [33]. The functional consequence of higher *EI* in the A-P orientation may also imply that the mechanoreceptors in the underlying follicle are more sensitive to flow changes in front of the animal, as opposed to above the animal, or may result in concomitant change in mechanoreceptor distribution and type around the hair shaft in the F-SC. Because many pinnipeds track prey in the water column, the A-P orientation of the vibrissae may be the most important in detecting and tracking hydrodynamic trails.

This is the first comparative study on the mechanical properties of pinniped mystacial vibrissae. Additional comparative research on the material properties of vibrissal hairshafts is needed to further elucidate pinniped vibrissal function. Other vibrissal fields in pinnipeds such as the rhinal and superciliary vibrissal fields were outside the scope of this study, but warrant further investigation. Our results indicate that flexural stiffness varies among species. Vibrissae exhibited higher *EI* values in the anterior-posterior orientation than the dorsal-ventral orientation, which was related to cross-sectional shape (ellipses) as measured by the major and minor axes that changed values of *I* while *E* remained constant. We suggest that the variation in flexural stiffness in pinniped vibrissae results in variation in resonance frequencies of hair shafts that interact with the beaded morphology to provide a greater resolution of hydrodynamic reception in phocid seals with beaded vibrissae. Recent research on pinniped vibrissae has focused on the differences in shape among some phocids, which demonstrate a sinusoidal beaded profile, and other phocids, otariids and odobenids, which have a smooth vibrissal profile [29,32]. The results of this study suggest that the mechanical properties of vibrissae may be just as important as the morphological profile in vibrissal function.
